# *Toxoplasma gondii* Infection Specifically Increases the Levels of Key Host MicroRNAs

**DOI:** 10.1371/journal.pone.0008742

**Published:** 2010-01-15

**Authors:** Gusti M. Zeiner, Kara L. Norman, J. Michael Thomson, Scott M. Hammond, John C. Boothroyd

**Affiliations:** 1 Department of Microbiology and Immunology, Stanford University School of Medicine, Stanford, California, United States of America; 2 Department of Cell and Developmental Biology, University of North Carolina, Chapel Hill, North Carolina, United States of America; 3 Lineberger Comprehensive Cancer Center, University of North Carolina, Chapel Hill, North Carolina, United States of America; Federal University of São Paulo, Brazil

## Abstract

**Background:**

The apicomplexan parasite *Toxoplasma gondii* can infect and replicate in virtually any nucleated cell in many species of warm-blooded animals; thus, it has evolved the ability to exploit well-conserved biological processes common to its diverse hosts. Here we have investigated whether *Toxoplasma* modulates the levels of host microRNAs (miRNAs) during infection.

**Methodology/Principal Findings:**

Using microarray profiling and a combination of conventional molecular approaches we report that *Toxoplasma* specifically modulates the expression of important host microRNAs during infection. We show that both the primary transcripts for *miR-17∼92* and *miR-106b∼25* and the pivotal miRNAs that are derived from *miR-17∼92* display increased abundance in *Toxoplasma-*infected primary human cells; a *Toxoplasma*-dependent up-regulation of the *miR-17∼92* promoter is at least partly responsible for this increase. The abundance of mature miR-17 family members, which are derived from these two miRNA clusters, remains unchanged in host cells infected with the closely related apicomplexan *Neospora caninum*; thus, the *Toxoplasma-*induced increase in their abundance is a highly directed process rather than a general host response to infection.

**Conclusions/Significance:**

Altered levels of *miR-17∼92* and *miR-106b∼25* are known to play crucial roles in mammalian cell regulation and have been implicated in numerous hyperproliferative diseases although the mechanisms driving their altered expression are unknown. Hence, in addition to the implications of these findings on the host-pathogen interaction, *Toxoplasma* may represent a powerful probe for understanding the normal mechanisms that regulate the levels of key host miRNAs.

## Introduction

The intracellular protozoan parasite *Toxoplasma gondii* is a ubiquitous pathogen of warm-blooded animals with approximately one to two billion humans infected [1]. While infections in immunocompetent adults are typically subclinical, infection persists for the life of the host, and serious disease can occur in fetal infections and in primary or recrudescent infection of immunocompromised persons. During host cell invasion, *Toxoplasma* uses a specialized set of secretory organelles to inject parasite-derived effector molecules into its host cell [2]. Some of these effectors are known to interfere with host cell signaling pathways and alter host defenses although the means by which they do this are not known [3]–[5]. *Toxoplasma* appears to block apoptotic responsiveness in its host cells at myriad points [6] and to co-opt host apoptotic signaling pathways as an environment-sensing mechanism [7]. *Toxoplasma-*infected cell cultures can also cede control of their cell cycle, progressing through G1/S and halting at G2/M and *Toxoplasma* attaches to, and invades host cells in S phase up to 4 times more efficiently than host cells in G1 [8]–[11]. In addition to these alterations in host cell signaling and cell cycle control, *Toxoplasma* specifically modulates host cell gene expression. Changes to the *Toxoplasma*-infected host transcriptome have been interrogated by expression-profiling and the data demonstrate that 24 hours post-infection, upwards of 15% of host mRNAs display altered abundance relative to uninfected cells [12]; similarly, quantitative analysis of the host proteome during *Toxoplasma* infection showed that the abundance of many host proteins is modulated in expression by *Toxoplasma* [13].

We have developed the hypothesis that some of these changes in host gene expression involve *Toxoplasma* co-opting host microRNAs (miRNAs), which are central components of an ancient post-transcriptional gene-regulatory mechanism that is highly conserved among the mammalian and avian hosts of *Toxoplasma*. MiRNAs are one of the most abundant classes of gene-regulatory molecules in the cell [14] and *in silico* target prediction suggests that miRNAs may be involved in the regulation of up to 30% of the human transcriptome [15]. Mature miRNAs are ∼23 nucleotide double-stranded RNA molecules that base-pair with target mRNAs. Most reports show that miRNAs act to negatively influence the translation and/or stability of target mRNAs [16], although under conditions of cell cycle arrest, some miRNAs appear to increase the translation of their target mRNAs [17]. Some closely related miRNAs are grouped into families by virtue of containing identical seed sequences (the first 6-8 nucleotides of their mature 5′ ends); miRNA seeds are instrumental in target mRNA discrimination [18] and so individual miRNAs grouped into the same family are believed to be functionally equivalent [19], [20].

**Figure 1:**
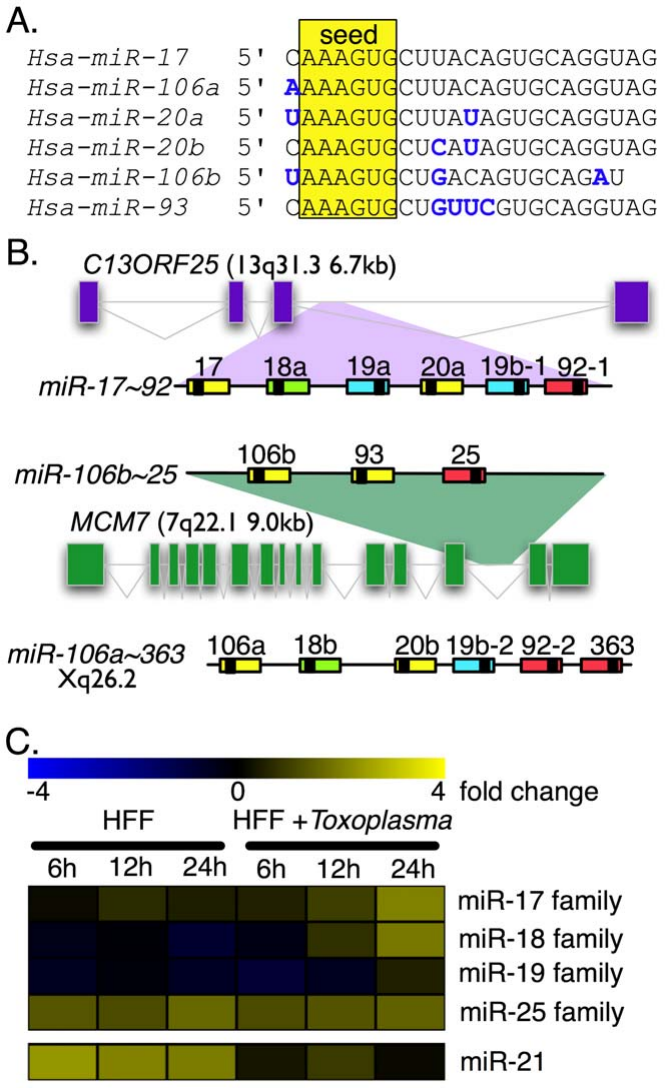
MiRNA microarray profiling reveals that Toxoplasma infection increases the levels of miR-17-, miR-18- and miR-19-family members. (A) Sequence alignment of miR-17 family members. The 6-nucleotide seed region that defines the miR-17 family is indicated in yellow. Nucleotide differences from the miR-17 sequence are shown in boldface blue type. (B) Genomic organization of the *miR-17∼92*, *miR-106b∼25* and *miR-106a∼363* clusters. Colors of each miRNA indicate the miRNA family to which each belongs; miR-17 = yellow; miR-18 = green; miR-19 = blue; miR-25 = red. miR-17∼92 and miR-106b∼25 are encoded in the 3rd intron of C13ORF25 and the 13^th^ intron of MCM7, respectively; for these genes, exons are indicated as boxes, and introns are lines. (C) Microarray profiling of host miRNAs during *Toxoplasma* infection. From left to right, columns are from individual microarrays hybridized with RNA extracted from uninfected HFFs at 6, 12 or 24 hours and with RNA samples derived from HFFs infected with *Toxoplasma* at 6, 12 or 24 hours. The data were normalized with the default settings in the Stanford Microarray Database software package. The scale of the heat-map is presented as fold-change from the median value. The rows are the averaged data from all probes that are predicted to hybridize to members of the indicated miRNA families; these families represent all miRNAs encoded by the *miR-17∼92*, *miR-106a∼363* and *miR-106b∼25* clusters. Shown for comparison is miR-21 which is a highly expressed miRNA in HFFs and is derived from an independent intergenic locus.

Although miRNAs were first identified due to their regulatory roles in sculpting metazoan development [21], [22], miRNA dysregulation also plays a central role in cancer and infection [20], [23]-[28]. The dysregulation of several miRNAs have been experimentally demonstrated to promote oncogenesis [20], [28]. Examples relevant to the present work are four families of miRNAs (miR-17, miR-18, miR-19 and miR-25) that are encoded by three paralogous loci; these related loci, which are *miR-17∼92, miR-106b∼25* and *miR-106a∼363* (see Figure 1B), produce primary transcripts that are post-transcriptionally processed to yield mature miR-17, miR-18, miR-19 and miR-25 family members. *MiR-17∼92* is also known by the synonym *OncomiR-1* due to the observed acceleration of lymphomagenesis when these miRNAs are over-expressed in Eµ-Myc mice [29].

Functionally, slight perturbations to the levels of mature *miR-17∼92*-derived miRNAs can have profound biological consequences; knock-in mice engineered to produce twice as much *miR-17∼92-*derived miRNAs as wildtype mice suffer lymphoproliferative phenotypes, autoimmunity and a shortened life-span linked to the loss of the PTEN tumor suppressor and the pro-apoptotic factor Bim, which are both validated targets of *miR-17∼92* [30]. Indeed, the *miR-17∼92* and *miR-106b∼25* clusters appear to attenuate apoptotic responsiveness by targeting several mRNAs encoding pro-apoptotic effectors and favor progression from G1 to S-phase by targeting mRNAs that encode negative regulators of the cell cycle [19], [20].

To investigate whether *Toxoplasma* infection drives changes to host miRNA levels, we profiled host miRNA levels during *Toxoplasma* infection with miRNA microarrays and confirmed these results by independent means. The data reveal numerous, *Toxoplasma*-specific changes to host miRNA levels and some of these changes are at least partly mediated at the transcriptional level.

## Results

### *Toxoplasma gondii* Alters MiRNA Levels in Host Cells

To investigate whether *Toxoplasma* infection alters the steady-state levels of host miRNAs, we profiled the levels of miRNAs extracted from primary human foreskin fibroblasts (HFFs) during a 24-hour *Toxoplasma* infection timecourse (where host RNA was extracted at 6-hours, 12-hours and 24-hours from mock- and from *Toxoplasma*-infected cells) using miRNA microarrays spotted with duplicate probes that should hybridize to all human, mouse and rat miRNAs included in miRbase v.8. When we compared hybridization intensities between arrays that were hybridized with RNA derived from uninfected HFFs and *Toxoplasma*-infected HFFs, the results demonstrated that at 24-hours post-infection, ∼14% of the spots displayed dissimilar hybridization intensities (Figure S1). We focused our attention on 18 microarray spots (9 closely related human and mouse probe sequences that were spotted in duplicate) that displayed comparable increases in hybridization intensities on arrays hybridized with RNA derived from infected HFFs (Figure S1, red boxes); these 18 spots contained probes that hybridized to members of the miR-17 family (the miR-17 family is composed of miR-17, miR-106a, miR-106b, miR-20a, miR-20b and miR-93; see Figure 1A for a sequence alignment of the miR-17 family). As miR-17 family members are co-transcribed with members of the miR-18 (Figure S1, blue box), miR-19 and miR-25 families, and are encoded in three separate paralogous loci (*miR-17∼92*, *miR-106a∼363* and *miR-106b∼25*; Figure 1B), we assembled a heat-map from our microarray data that contains the averaged fold-change values for all probes that hybridized to members of the miR-17, miR-18, miR-19 or miR-25 families (18, 4, 4, and 4 probes, respectively; Figure 1C). When evaluating these data it is important to note that within these individual miRNA families, mature miRNAs have nearly identical nucleotide sequences, and are virtually indistinguishable from the other members of their own miRNA family by microarray, northern blotting, and conventional primer-extension analyses.

The microarray profiling data showed that the miR-17 family and the miRNA families that are co-transcribed with it were generally more abundant in RNA samples from *Toxoplasma*-infected cells and that the abundance of these miRNAs marginally increased at 6-hours post-infection and showed greater increases at 12-hours and 24-hours post-infection (Figure 1C). MiR-21 is a highly abundant unrelated miRNA in HFFs that is derived from an independent intergenic locus and is included in the microarray heat-map and subsequent analyses for comparison.

To validate the microarray data and to specifically measure the effects of *Toxoplasma* infection on miRNAs encoded by *miR-17∼92*, *miR-106a∼363* and *miR-106b∼25*, we took two orthogonal approaches. First, we performed high-resolution northern blot analysis for miR-17. Consistent with the microarray data, the northern blot results showed that, collectively, the level of mature miR-17 family members increased ∼2.7-fold in *Toxoplasma*-infected HFFs relative to uninfected HFFs (Figure S2). The data also showed that the pre-miR-17 signal was a small fraction of the hybridization signal observed for mature miR-17, suggesting that the contribution of pre-miR-17 to the observed miRNA microarray hybridization intensity was negligible, an interpretation that is consistent with previous data [31].

**Figure 2:**
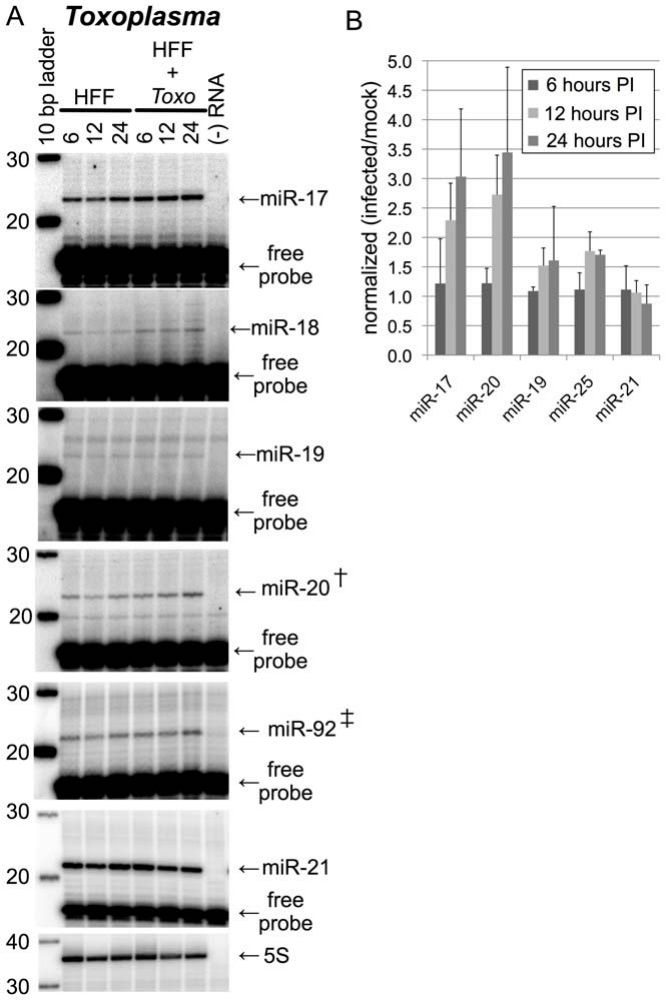
Primer-extension analysis shows that Toxoplasma infection specifically increases levels of host miR-17, miR-18, miR-19 and miR-25 family members. (A) Total RNA samples were extracted from HFFs during a 24-hour timecourse of *Toxoplasma-* infection, and subject to primer extension analysis. From left to right, template RNAs were derived from uninfected HFFs at 6, 12 or 24 hours, or from infected HFFs at the same time points. Shown are representative autoradiographs for each primer-extension probe; all reactions were performed on biological triplicates. All probes were also extended in the absence of template RNA (‘- RNA’ lanes) to identify any primer-extension artifacts associated with each probe. Primer-extension products are shown with arrows and labeled. (†) miR-20 is a member of the miR-17 family; (‡) miR-92 is a member of the miR-25 family. Excess free probe is indicated. The size marker is a 10bp ladder. (B) A graphical summary of the primer-extension data in (A). Each RNA sample was subject to primer-extension with the indicated miR-specific probe and band-intensities were quantified by phosphorimager analysis. These values were normalized to values obtained from an independent primer-extension of each RNA sample with a 5S rRNA probe. The 5S rRNA-normalized infected/uninfected ratios for each timepoint are plotted and error bars are the standard deviation between three biological replicates that were performed for each timepoint.

The second approach used to follow up on the microarray results was primer-extension analysis with primers specific for each miR family. The raw data shown in Figure 2A confirmed that the levels of mature miR-17 family members increased as a function of time in RNA samples derived from *Toxoplasma-*infected HFFs. Primer-extensions performed with probes specific for 5S rRNA and miR-21 gave very similar patterns to each other with no significant effect of *Toxoplasma* infection (Figure 2A). MiR-18, miR-19 and miR-25 family members also showed an increase in abundance in RNA samples derived form *Toxoplasma*-infected HFFs (Figures 2C). A graphical representation of all replicate primer-extensions normalized to 5S rRNA is plotted in Figure 2B. Upon *Toxoplasma* infection, miR-17 family members collectively increased ∼3-fold, and miR-19 and miR-25 family members increased ∼1.5-fold. When normalized to 5S rRNA, miR-18 also increased upon *Toxoplasma* infection, but the measurements for the miR-18 primer-extensions were complicated by low signal and high background (see Figure 2A), making its measurement less reliable; it was therefore not included in Figure 2B. Similar primer-extension analyses were performed with probes for the miR-17 family and 5S rRNA on RNA samples derived from two *Toxoplasma*-infected mouse embryonic fibroblast lines (C57/B6/129 [32] and NIH 3T3 [33]) and from a human B-cell line (Raji [34]); the results of these experiments were consistent with the primary HFF results detailed above (data not shown).

The data from the miRNA microarray profiling and primer-extension analysis were generally in agreement except in one instance. The microarray data (Figure 1B) indicated that the miR-25 family of miRNAs did not appreciably increase in arrays hybridized with RNA derived from *Toxoplasma-*infected cells at the 24 hour timepoint whereas primer-extension for miR-92 (the miR-25 paralog derived from the *miR-17∼92* locus) indicated a modest but reproducible increase (Figure 2B). The Cy5 channel intensities of the miR-25 family probe spots were near the upper limits of the dynamic range of the microarrays, which likely obscured the signal increase seen in the primer-extensions. Excluding this discrepancy, the primer-extension data closely approximate the results of the northern blot and miRNA microarray profiling and confirm that *Toxoplasma* increases the levels of mature miR-17, miR-18, miR-19 and miR-25 family members in infected human cells.

### Increases in MiR-17 Family Members Are a Specific Response to Toxoplasma Infection

**Figure 3:**
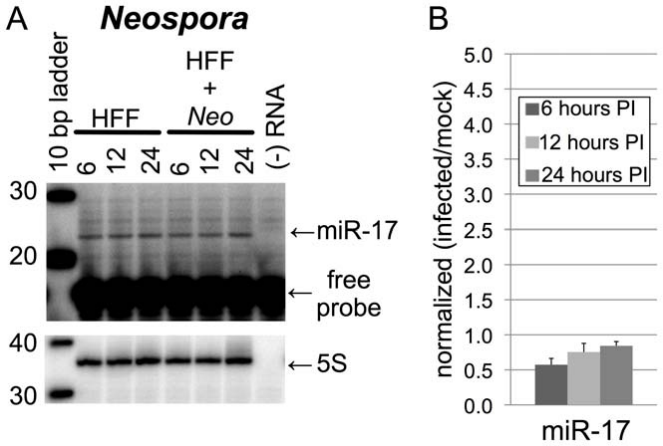
Neospora infection does not alter the levels of miR-17 family members. (A) Total RNA samples were extracted from HFFs during a 24-hour timecourse of *Neospora-*infection, and subject to primer extension analysis. From left to right, template RNAs were derived from uninfected HFFs at 6, 12 or 24 hours, or from infected HFFs at the same time points. Shown are representative autoradiographs for primer-extension with the miR-17 and 5S probes; reactions were performed on biological triplicates. miR-17 and 5S probes were also extended in the absence of template RNA (‘- RNA’ lanes) to identify any primer-extension artifacts associated with each probe. Primer-extension products are shown with arrows and labeled. Excess free probe is indicated. The size marker is a 10bp ladder. (B) A graphical summary of the primer-extension data in (A); the scale has been preserved for comparison with Figure 2B. RNA samples were subject to primer-extension with the miR-17 probe and band-intensities were quantified by phosphorimager analysis. These values were normalized to values obtained from an independent primer-extension of each RNA sample with a 5S rRNA probe. The 5S rRNA-normalized infected/uninfected ratios for each timepoint are plotted and error bars are the standard deviation between three biological replicates that were performed for each timepoint.

To investigate whether the observed increase in host miR-17 levels is a specific response to *Toxoplasma* infection or a general response of apicomplexan-infected host cells, we assayed the levels of host miR-17 in HFFs infected with *Neospora caninum*. *Neospora* and *Toxoplasma* are very closely related coccidians, have similar asexual growth cycles, and are morphologically almost indistinguishable [35]. Although *Neospora* is not a human pathogen and has a far more restricted host range than *Toxoplasma*, *Neospora* readily infects HFFs and grows with kinetics comparable to *Toxoplasma* in these cells [36]. To assess whether *Neospora* infection changes the levels of host miR-17 family in a manner similar to *Toxoplasma* infection, we performed primer-extension analysis on RNA samples derived from HFFs comparably infected with *Neospora*. The results showed that *Neospora* infection did not significantly alter the levels of host miR-17 family members using either a miR-17 probe (Figure 3A and Figure 3B), or miR-20 probe (data not shown) at any time-point in our assay. We conclude that the increased level of miR-17 family members seen in *Toxoplasma-*infected cells is *Toxoplasma*-specific and is not a general host response to apicomplexan infection.

### Toxoplasma- and Neospora-Infected HFFs Show Elevated Levels of Pri-MiRNAs Encoding MiR-17 Family Members

**Figure 4:**
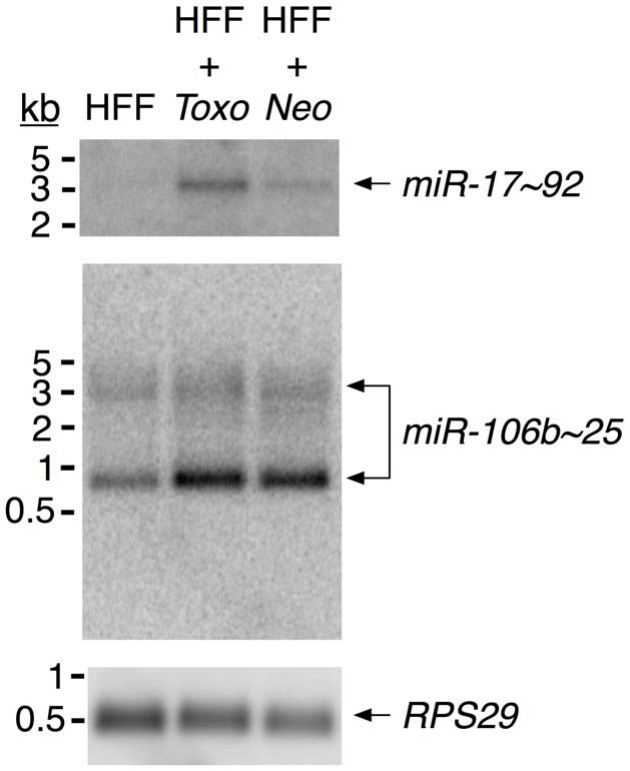
Toxoplasma-infected HFFs have elevated levels of pri-miR-17∼92 and pri-miR-106b∼25. Total RNA was isolated from uninfected HFFs (‘HFF’ lane), or HFFs infected for 24 hours with *Toxoplasma* (‘HFF+*Toxo*’ lane) or *Neospora* (‘HFF+*Neo*’ lane). From top to bottom panels, probes are: *miR-17∼92*, *miR-106b∼25* and *RPS29*. The labeling, hybridization, washes, and exposures were performed identically for each probe. Sizes (kb) are derived from a co-resolved RNA ladder.

To investigate whether *Toxoplasma* manipulates the levels of pri-miRNAs encoding miR-17, miR-18, miR-19 and miR-25 family members, and to query which of the paralogous clusters encoding miR-17 family members are manipulated by *Toxoplasma*, we performed northern blot analysis on *pri-miR-17∼92*, *pri-miR-106a∼363* and *pri-miR-106b∼25* (Figure 4). This was done using probes that hybridize to sequences that are unique to each cluster (sequences that encode the pre-miRNA hairpins are not recognized by the probes; sequences provided in Figure S3), thereby allowing us to discriminate between the three paralogous clusters. The hybridization products with different probes were quantified by phosphorimager analysis, normalized to the signal for the constitutively expressed *RPS29* to correct for loading variation, and expressed as a fraction of the signal derived from uninfected HFFs for each probe. A parallel analysis was performed using a probe for *AldoA* as a loading control and yielded comparable numbers to those obtained by normalization with *RPS29* (data not shown).

Figure 4 shows that hybridization with the pri-miR-17∼92 probe yields a ∼3.2kb band which is a size previously published for the pri-miRNA derived from this cluster [37]. The level of *pri-miR-17∼92* in HFFs infected with *Toxoplasma* relative to uninfected HFFs was ∼3.6-fold greater whereas HFFs infected with *Neospora* showed no such increase (∼0.7-fold relative to uninfected HFFs). When the membrane was stripped and hybridized with a pri-miR-106b∼25 probe, infection with either *Toxoplasma* or *Neospora* showed a major band at ∼850nt, and this band demonstrated an increase of ∼2.5-fold and ∼2.6-fold, respectively, relative to uninfected HFFs (Figure 4). There are no previous reports of *pri-miR-106b∼25* northern blots and so, while we cannot be certain that this band corresponds to *pri-miR-106b∼25* or to a splice variant of *MCM7*, it was consistently seen in multiple experiments and is within the size range expected for this molecule. No reproducible *pri-miR-106a∼363* hybridization products were visible (data not shown).

These results suggest that the mature miR-18 and miR-19 family members that increase upon infection with *Toxoplasma* are derived from *miR-17∼92*; although *pri-miR-106b∼25* is also increased in *Toxoplasma*-infected cells, *miR-106b∼25* does not encode miR-18 and miR-19 family members and, consistent with previous reports [32], [37], no *pri-miR-106a∼363* was detectable.

### Toxoplasma Infection Increases Transcription from the Pri-MiR-17∼92 Promoter

**Figure 5:**
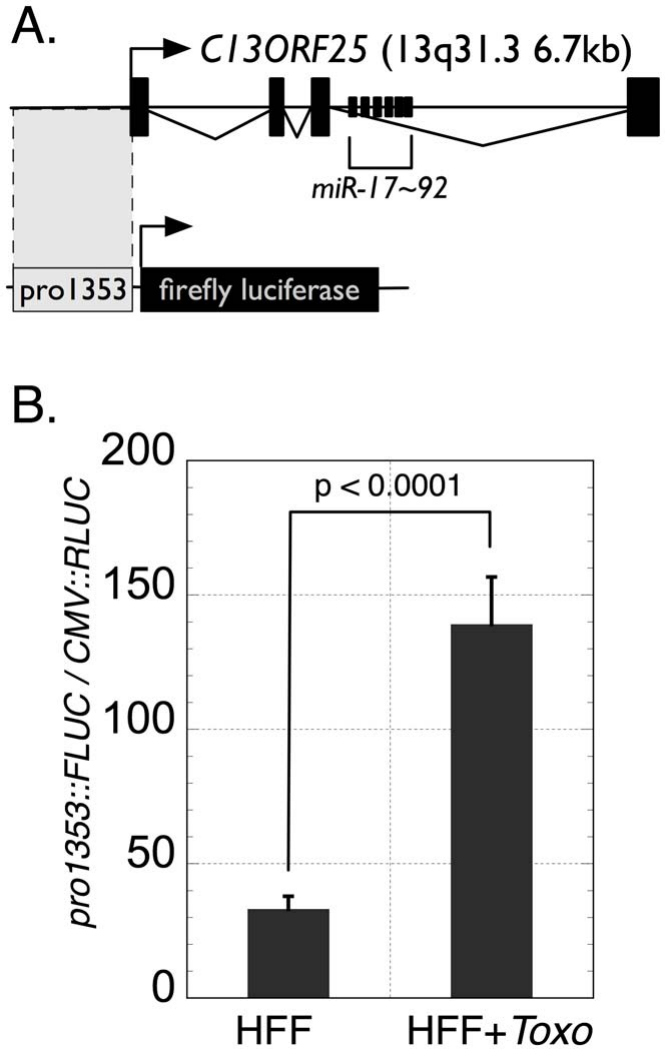
Toxoplasma infection increases pri-miR-17∼92 transcription. (A) Diagram of the location of the *pro1353* fragment and its position relative to the transcribed portion of *C13ORF25*. Transcription start sites of *C13ORF25* and *pro1353::FLUC* are shown by bent arrows. The *pro1353* fragment overlaps the 5′-end of the *C13ORF25* EST by 10 nucleotides [38]. (B) *pro1353::FLUC*/*CMV::RLUC* ratios for uninfected HFFs or *Toxoplasma*-infected HFFs (HFF+*Toxo*) are plotted. Shown are five biological replicates for each condition from a representative experiment.

To investigate whether the *Toxoplasma* infection-dependent increase in *pri-miR-17∼92* abundance is the result of increased transcription from this locus, we performed a dual luciferase assay with a reporter plasmid [38] containing a 1.3kb fragment located upstream of *C13ORF25* fused to a promoter-less firefly luciferase (FLUC) cassette (*pro1353::FLUC*; see Figure 5A); this 1.3kb upstream fragment of *C13ORF25* overlaps the 5′ terminus of the *C13ORF25* expressed sequence tag (EST) by 10 nucleotides and has been previously shown to drive firefly luciferase expression [38]. The *pro1353::FLUC* plasmid was co-transfected into HFFs with a plasmid that constitutively expresses *Renilla* luciferase (RLUC; pRL4.75). The transfected cells were either mock- or *Toxoplasma*-infected and the ratio of FLUC/RLUC was determined at 18 hours post-infection.

The data demonstrate that *Toxoplasma*-infected HFFs show a 4.3-fold increase in FLUC/RLUC relative to mock-infected HFFs (p = 8.8×10^−5^; Figure 5B). These data strongly suggest that the increased level of *pri-miR-17∼92* seen in *Toxoplasma*-infected HFFs is a result of increased *miR-17∼92* transcription rather than alterations in the stability of *pri-miR-17∼92*, as only 10 nucleotides from the *pri-miR-17∼92* transcript are present in the luciferase reporter construction.

## Discussion

The results presented here demonstrate that *Toxoplasma* infection specifically drives an increase of 2–3 fold in the levels of mature *miR-17∼92*-derived miRNAs in primary human foreskin fibroblasts. The mature miRNAs encoded by *miR-17∼92* and its paralog *miR-106b∼25* play important roles in mammalian biology. *MiR-17∼92* is crucial in development, as mice harboring a homozygous deletion of *miR-17∼92* die shortly after birth due to pulmonary and cardiac defects and develop abnormal B-cell lymphocytes [32]. In adult animals, *miR-17∼92* and *miR-106b∼25* have been shown to influence the functionally intertwined pathways of apoptosis and G1/S cell cycle progression by targeting multiple components of each pathway [20]. The importance of these functions have been demonstrated by showing that retroviral overexpression of a cassette containing miR-17, miR-18 and miR-19 in mice results in c-Myc-induced lymphoma [29]. More subtly, transgenic mice that have been engineered to overexpress *miR-17∼92* by 2-fold in lymphocytes develop lymphoproliferative disease and autoimmunity and die prematurely, phenotypes that were shown to be partially a consequence of the translational repression of PTEN and Bim [30]. Hence, even relatively modest increases in the levels of *miR-17∼92*, of a magnitude less even than seen here with *Toxoplasma* infection, have profound biological consequences *in vivo*.

The ultimate, downstream target of these changes in miRNAs, in terms of benefit to the parasite, is not clear; in the set of HFF mRNAs that decrease in abundance upon *Toxoplasma* infection [5], there is no significant enrichment for mRNAs containing predicted *miR-17∼92* or *miR-106b∼25* binding sites (data not shown). This lack of apparent enrichment could be due to the very large number of changes induced by *Toxoplasma* through other means; for example, many transcription factors are up-regulated in *Toxoplasma*-infected cells, which significantly increases the background for this kind of bioinformatics analysis. It could also signify that the critical, intended effect of modulating these miRNAs is on translation rather than transcription [37], [39].

To understand the role of increased *miR-106b∼25* and/or *miR-17∼92* in *Toxoplasma* infection we took several approaches. First, we infected primary mouse fibroblasts (MEFs) harboring a *miR-17∼92* deletion [32]. The results showed no reproducible difference in *Toxoplasma* growth in such cells relative to wild type controls (data not shown). Second, concurrent analyses using locked nucleic acid-mediated knockdown of *miR-17∼92-* and *miR-106b∼25*-derived miRNAs was performed using a wide range of conditions in mouse fibroblasts and HFFs. The results showed no reproducible effects of knockdown of these miRNAs on the ability of *Toxoplasma* to infect, grow within and lyse the host cell *in vitro* (data not shown). This absence of an effect is consistent with the fact that while many of these miRNAs are extremely important in the animal [30], [32], they are largely dispensable for simple, *in vitro* growth of cell lines (*miR-17∼92* knockout MEFs have no obvious *in vitro* growth defect and, in general, most commonly used transformed cell lines dramatically overexpress *miR-17∼92*). Extensive Affymetrix microarray profiling of *Toxoplasma*-infected wildtype- or *miR-17∼92*-knockout MEFs showed no statistically significant differences in mouse mRNA levels 24h post infection (data not shown).

To address what the important biological target(s) of these miRNAs are in the context of *Toxoplasma* infection, *in vivo* studies will be needed. Unfortunately, as homozygous *miR-17∼92* knockout mice are not viable [32] dissecting the impact of these genes on *Toxoplasma* pathogenesis is technically difficult. The situation is further complicated by the fact that both *pri-miR-17∼92* and *pri-miR-106b∼25* are increased upon *Toxoplasma* infection and these two clusters are believed to be partially functionally redundant. Ultimately, a conditional knockout of *miR-17∼92* in a *miR-106b∼25*-null background will be necessary to score the full biological role(s) of these miRNAs in *Toxoplasma*-infected animals.

Whatever the biological role of these miRNAs in *Toxoplasma* infection, the results presented here demonstrate that increases in *miR-17∼92-*derived miRNAs are a specific response to *Toxoplasma* infection. During such infection only a subset of miRNAs are affected and an increase in *miR-17∼92*-derived miRNAs is not seen when cells are similarly infected with the closely related coccidian *Neospora*. To our knowledge this represents the first report of a pathogen that specifically increases the levels of *miR-17∼92* and/or *miR-106b∼25.* Thus, in addition to the importance of understanding the host-pathogen interaction, *Toxoplasma* and *Neospora* could prove extremely useful as probes for dissecting the biogenesis and function of miRNAs in normal, uninfected human cells.

The levels of *miR-17∼92*-derived miRNAs reported here are at least partially due to a *Toxoplasma*-dependent increase in *pri-miR-17∼92* transcription. The transcription of *pri-miR-17∼92* has been shown to be positively regulated by E2F3 [38] and c-Myc [37] transcription factors, and *pri-miR-106b∼25* is positively regulated by E2F1 [40]. Expression-profiling of *Toxoplasma*-infected HFFs [5] demonstrated that there are modest increases in the levels of *E2F3* (+1.4-fold), *c-Myc* (+1.9-fold) and *E2F1* (+1.5-fold) in *Toxoplasma-*infected HFFs relative to uninfected HFFs at 24h post-infection. It is, therefore, probable that the *Toxoplasma*-dependent increase in the levels of *pri-miR-17∼92* and *pri-miR-106b∼25* are at least in part due to such changes in host transcription factors.

Whether the increases in *miR-17∼92*-derived miRNAs are mediated by c-Myc, E2F or another pathway, the identity of the parasite factor(s) that drive these changes are unknown. These changes could involve one or more of the recently reported effectors that the parasite injects into the host cell during infection. These effectors include protein phosphatases [41] and protein kinases [4], [5], one of which has been shown to influence *Toxoplasma* virulence by dramatically altering host transcription through interacting with STAT3 and STAT6 functions [5]. Whether this or one of the other injected *Toxoplasma* effectors is responsible for the alterations in host *miR-17∼92* and *miR-106b∼25* expression, and what role these changes play on the host-pathogen interaction *in vivo* are the focus of on-going work.

## Materials and Methods

### Parasite Strains and Cell Culture

Parasites were propagated in primary HFFs and Vero cells using standard cell culture techniques as described previously [12]. Manipulations and host cell passage histories are expanded in the Detailed Materials and Methods (Appendix S1).

### RNA Isolation

RNA was extracted with TRIzol (Invitrogen), and fractionations were performed with the miRvana™ miRNA isolation kit (Ambion) or the Oligotex kit (Qiagen) according to the manufacturer's instructions. Expanded RNA methods are provided in the Detailed Materials and Methods (Appendix S1).

### MicroRNA Microarray Profiling

Spotted oligonucleotide mirVana™ miRNA Probe Set version 2 (Ambion) miRNA microarrays were purchased from the Stanford Functional Genomics Facility. Extracted miRNAs were 3′ end labeled with pCp-Cyanine dyes (pCp-Cy3 for common reference, which was a mixture of all hybridized samples and pCp-Cy5 for each individual experimental sample). pCp-Cyanine dyes were synthesized at the Stanford PAN facility. Arrays were hybridized, washed and processed according to the manufacturer's instructions (Ambion). Expanded microarray methods are provided in the Detailed Materials and Methods (Appendix S1).

### Northern Blotting

MiRNA and pri-miRNA northern blotting were performed essentially as previously reported [37]. For pri-miRNA northern blotting, 100 µg total RNA samples were fractionated with Oligotex (Qiagen) according to the manufacturer's instructions prior to 1% agarose/formaldehyde separation and capillary transfer. Expanded northern blot methods are provided in the Detailed Materials and Methods (Appendix S1).

### Primer-Extension Analysis

MiRNA primer extensions were performed in a manner similar to a previous report [23]. Expanded primer extension methods provided in the Detailed Materials and Methods (Appendix S1).

## Supporting Information

Figure S1 miRNA microarray profiling data of Toxoplasma-infected primary human foreskin fibroblasts. Columns are individual microarrays hybridized with labeled, size-fractionated RNA derived from mock infected HFFs or Toxoplasma-infected HFFs at the indicated time-points. Heatmap scale is from −3 to +3, and these values reflect the log2-transformed ratios of the hybridization intensities of Cy5-labeled sample/Cy3-labeled common reference for each array. Common reference RNA was a pooled mixture of RNAs from all samples. Data was filtered in SMD according to the default settings, and only spots that were called ‘present’ on 80% of the arrays were included in the heatmap. Log2-transformed ratios of the hybridization intensities of sample/common reference for each spot on each array were hierarchically clustered (Euclidean) in MEviewer (MeV4.2; TIGR) by arrays and to genes. Red boxes are miR-17 family members; blue boxes are miR-18 family members. has = human, mmu = mouse, rno = rat, ambi = proprietary Ambion probe sequences.
(9.27 MB TIF)

Figure S2 Northern blot analysis demonstrates that Toxoplasma infection results in increased levels of mature miR-17 family members. RNA samples derived from uninfected HFFs (- lanes), and from HFFs infected with Toxoplasma for 24h (+ lanes) were resolved through a 10% acrylamide/8M urea gel, transferred to nylon membrane, and hybridized to miR-21, miR-17 and U6 oligonucleotide probes (corresponding probes are indicated underneath autoradiographs). The U6 snRNA hybridization is shown as a loading control. Putative pre-miRNA bands and mature miRNA bands are indicated with arrows. A co-electrophoresed 10nt ladder is shown for nucleotide size comparison.
(0.42 MB TIF)

Figure S3 Probe sequences used in this study. All oligonucleotides are listed from 5′ to 3′. In ‘notes’ column, target nucleotide positions are provided for all primer-extension probes. The forward and reverse primers used in PCR amplification of the pri-miR-17∼92, pri-miR-106a∼363, pri-miR-106b∼25, AldoA, MCM7 and RPS29 probes are shown.
(0.33 MB DOC)

Appendix S1 Detailed Materials and Methods. This Supplemental Information file contains expanded Materials and Methods from the manuscript
(0.06 MB DOC)
