# Supplementary figures and images for: Toxoplasma gondii Infection Specifically Increases the Levels of Key Host MicroRNAs
Source: PLoS One. 2010 Jan 15;5(1):e8742. doi: 10.1371/journal.pone.0008742 (PMC2806928; doi:10.1371/journal.pone.0008742)

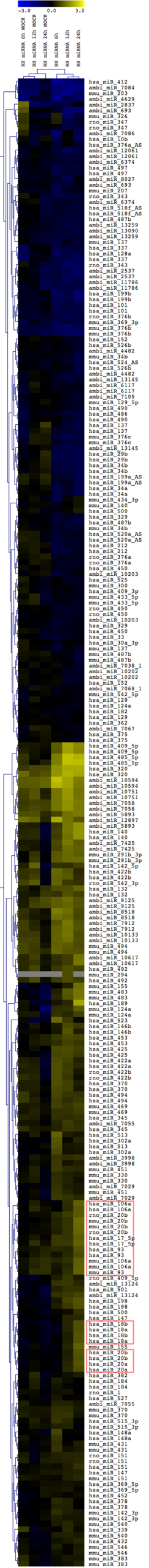

Supplement: Figure S1 — miRNA microarray profiling data of Toxoplasma-infected primary human foreskin fibroblasts. Columns are individual microarrays hybridized with labeled, size-fractionated RNA derived from mock infected HFFs or Toxoplasma-infected HFFs at the indicated time-points. Heatmap scale is from −3 to +3, and these values reflect the log2-transformed ratios of the hybridization intensities of Cy5-labeled sample/Cy3-labeled common reference for each array. Common reference RNA was a pooled mixture of RNAs from all samples. Data was filtered in SMD according to the default settings, and only spots that were called ‘present’ on 80% of the arrays were included in the heatmap. Log2-transformed ratios of the hybridization intensities of sample/common reference for each spot on each array were hierarchically clustered (Euclidean) in MEviewer (MeV4.2; TIGR) by arrays and to genes. Red boxes are miR-17 family members; blue boxes are miR-18 family members. has = human, mmu = mouse, rno = rat, ambi = proprietary Ambion probe sequences. (9.27 MB TIF) [file pone.0008742.s001.tif]

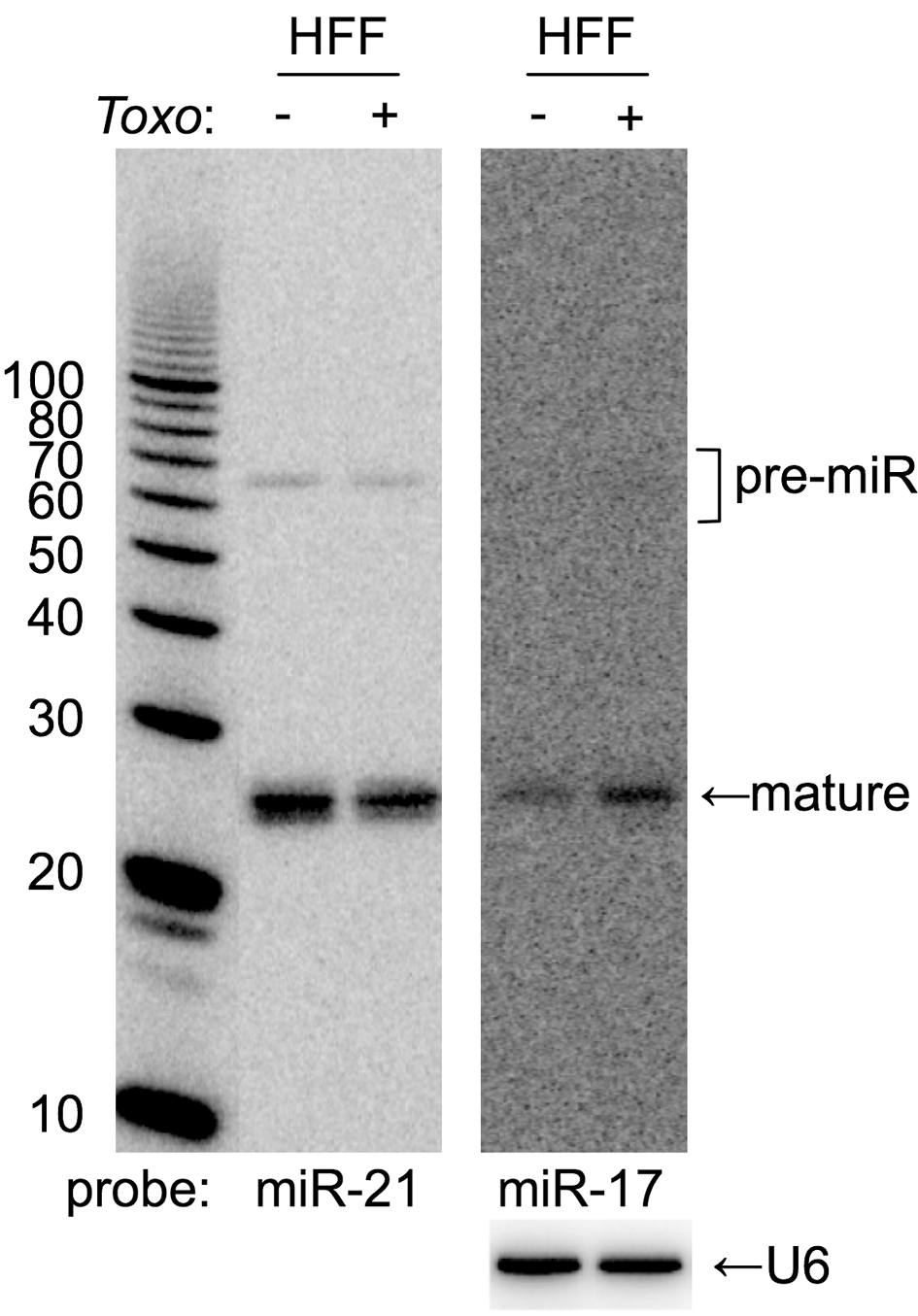

Supplement: Figure S2 — Northern blot analysis demonstrates that Toxoplasma infection results in increased levels of mature miR-17 family members. RNA samples derived from uninfected HFFs (- lanes), and from HFFs infected with Toxoplasma for 24h (+ lanes) were resolved through a 10% acrylamide/8M urea gel, transferred to nylon membrane, and hybridized to miR-21, miR-17 and U6 oligonucleotide probes (corresponding probes are indicated underneath autoradiographs). The U6 snRNA hybridization is shown as a loading control. Putative pre-miRNA bands and mature miRNA bands are indicated with arrows. A co-electrophoresed 10nt ladder is shown for nucleotide size comparison. (0.42 MB TIF) [file pone.0008742.s002.tif]
