# Supplementary material for: Toxoplasma gondii Infection Specifically Increases the Levels of Key Host MicroRNAs
Source: PLoS One. 2010 Jan 15;5(1):e8742. doi: 10.1371/journal.pone.0008742 (PMC2806928; doi:10.1371/journal.pone.0008742)
Supplement: Appendix S1 — Detailed Materials and Methods. This Supplemental Information file contains expanded Materials and Methods from the manuscript (0.06 MB DOC) [file pone.0008742.s004.doc]

***Detailed Materials and Methods***

*Parasite strains and cell culture*. Tachyzoites of *Toxoplasma gondii* (RH88 strain) and *Neospora caninum* (NC1 strain) were used in this study. RH88 and NC1 were maintained *in vitro* by serial passage on subconfluent monolayers of Vero cells (ATCC # CRL-2783) at 37°C with 5% CO_2_ in CDMEM [DMEM (GIBCO); 10% fetal calf serum (GIBCO); 2mM glutamine; 50µg/ml penicillin; 50µg/ml streptomycin]. This cell culture medium was also used for passage of primary HFFs (all primary HFFs were used between passages 7-10), NIH 3T3 MEFs[1], Raji B-cells[2] and for primary MEFs harboring *loxP*-flanked *miR-17~92* loci[3]; these MEFs were a generous gift from A. Ventura and T. Jacks (Massachusetts Institute of Technology).

For infection timecourse experiments (Figures 1, 2, S1) 150mm dishes 70% confluent with Vero cells were infected with the indicated parasites. When parasites reached the 64-128 parasites/vacuole stage the infected Vero monolayers and uninfected control plates were washed twice in DMEM then scraped and lysed by repeated passage through 27 gauge syringe needles. Parasite numbers were quantified using a hemocytometer. The following steps were performed for both mock-infected and infected cultures. Cell debris was removed by centrifuging for 1 minute at 700 RPM; supernatant from the low speed spin was decanted and centrifuged for 10 minutes at 2000 rpm in a tabletop centrifuge at room temperature, and the medium was aspirated. Parasite pellets were resuspended in 40ml DMEM and centrifuged in a tabletop centrifuge for 10 minutes at 1400 rpm at room temperature, and the medium was aspirated again; this last step was repeated two more times to minimize carryover.

The parasite pellet and mock-infected Vero cell debris pellet were resuspended in CDMEM and added to a fresh monolayer of confluent, recently passed primary HFFs in 150mm dishes at an MOI of 5 (6 plates per time-point; three biological replicate time-points for primer-extensions). At 4 hours post-infection, the medium was replaced with fresh CDMEM prewarmed to 37°C. At 6 hours post-infection the medium from biological replicate sets of mock + infected plates was aspirated, the cells rinsed once with 1x PBS, and 10 ml of TRIzol (Invitrogen) containing 1mg/ml Glycogen (Roche) was added to each plate. TRIzol-solublized cell lysates were scraped and passaged once through an 18 gauge syringe needle, then stored at -80°C until the 12 and 24 hour infection plates were processed. In a parallel set of experiments, a mixture of all mock-infected and infected time-points were pooled and labeled with Cy3 for use as a common reference for all miRNA microarray profiling experiments (see below).

*RNA methods.* The TRIzol-solublized frozen samples were warmed to room temperature, and total RNA was extracted according to the manufacturer's instructions. This total RNA from TRIzol extraction was used in all primer-extension analyses and high-resolution northern blotting experiments. For pri-miRNA northern blot analysis, 100µg of each TRIzol-extracted total RNA derived from mock infected HFFs, or HFFs infected with *Neospora* or *Toxoplasma* was fractionated by Oligotex chromatography (Qiagen); see below for details. For the miRNA microarray profiling experiment, a set of total TRIzol RNA pellets from an infection timecourse was fractionated into large (>200 nucleotide) and small (<200 nucleotide) fractions using the miRvana kit (Ambion) according to the manufacturer's instructions. RNA samples were stored in siliconized 1.7 ml tubes (Axygen Maxymum Recovery) at -80°C until used.

*Luciferase assays.* 1µg of *pro1353::FLUC* plasmid and 25ng of pRL4.75 (*CMV::RLUC*; Promega) were co-transfected into each well of a 12-well plate seeded with 80% confluent HFFs using Lipofectamine LTX (Invitrogen) according to the manufacturer’s instructions. At 15 hours post-transfection, <50% lysed cultures of *Toxoplasma gondii* RH tachyzoites were scraped and syringe lysed from HFFs, washed and counted by hemocytometer. At T=0, either washed supernatants from scraped and syringe-lysed uninfected HFFs (processed in parallel to counted RH) were added to ‘uninfected HFF’ wells, or 1x10^6^ RH were added to each well (MOI=5) of HFF+*Toxo* wells. At T=18h PI, medium was aspirated and cells were rinsed once with 1x PBS. Cells were then processed for measurement of luciferase activity with the dual luciferase assay kit (Promega) according to the manufacturer’s instructions on a Moonlight 2010 luminometer (Analytical Luminescence Laboratory). Luciferase assays were performed with five biological replicates of each condition, and each experiment was repeated three times. Standard deviations were calculated and a two-tailed Students t-test was performed. Activity was defined as Firefly/Renilla ratio, and these ratios were plotted with KaleidaGraph (Synergy Software).

*pCp-Cy3 and pCp-Cy5 synthesis.* Cyanine 3-Cytidine 3’,5’-bisphosphate (pCp-Cy3) and Cyanine 5-Cytidine 3’,5’-bisphosphate (pCp-Cy5) were synthesized at the Stanford PAN facility (http://cmgm.stanford.edu/pan/). All reagents were purchased from Glen Research. pCp-Cy3 and pCp-Cy5 were synthesized on a 1µmol scale Universal II support. The sequence 5'-XCYT-3' was programmed into the oligonucleotide synthesizer, where ‘X’ was the reservoir for the chemical phosphorylation reagent (Cat#10-1900), ‘Y’ was the reservoir containing Cy3- (Cat#10-5913) or Cy5 (Cat#10-5915) phosphoramidites, and ‘T’ was a place saver for the support. The pCp-Cy3 and pCp-Cy5 were synthesized in trityl-off mode.  After synthesis the support was rinsed with acetonitrile, dried with Argon, and the Cytidine was deprotected. The pCp-Cy3 and pCp-Cy5 were cleaved from the support by addition of 1 ml 2 M NH_3_ in anhydrous methanol for 1 hour at room temperature. After cleavage, 1 ml ammonium hydroxide was added for 4 hours at room temperature, the pCp-Cy3 and pCp-Cy5 were dried in a Speed-Vac, resuspended in 1 ml 1 M tetra-*N*-butylammonium fluoride in tetrahydrofuran, vortexed, and incubated for 6 hours at room temperature to remove the 2'-*O*-protection group. 1 ml 0.1 M triethylammonium acetate (TEAA) was added, and the solution was dried in a Speed-Vac until volume was reduced by half. The solution was loaded onto a conditioned RP cartridge, and was washed with 5 ml 0.1 M TEAA followed by 5 ml RNase free water to remove salts. The pCp-Cy3 and pCp-Cy5 were eluted by the addition of 2 ml 50% acetonitrile in RNase free water, and dried in a Speed-Vac. pCp-Cy3 and pCp-Cy5 were resuspended in RNase free water, and concentration was determined by Nanodrop spectrophotomery (Nanodrop).

*microRNA microarray profiling.* miRNA microarrays were purchased from the Stanford Functional Genomics Facility (http://microarray.org/sfgf/). The Ambion mirVana™ miRNA Probe Set version 2 was printed on SCHOTT Nexterion® Slide E microarray slides with Majer 17-4 ss print tips. Each microarray contains 668 spots printed in duplicate, comprising 328 human (hsa) miRNAs, 154 proprietary Ambion probes, 113 mouse (mmu) and 45 rat (rno) miRNAs that are non-identical orthologs of human miRNAs or unique and 28 empty control spots. We obtained a set of sequentially printed arrays; print quality and spot-to-spot intra-array normalization were assessed by staining control arrays distributed evenly through the sequentially numbered set of arrays with POPO-3-iodide (Invitrogen) according to the manufacturer’s instructions. POPO-3-iodide-stained arrays were unsuitable for subsequent hybridization, as after dye-stripping, residual POPO-3-iodide interfered with Cy5 signal quantification when hybridized with Cy5-labeled miRNA samples. 3.5 µg of small RNA from each timepoint and 3.5 µg of common reference small RNA fractions were individually dephosphorylated for 1 hour at 37°C with 15 units of Arctic Phosphatase (New England Biolabs) in 1x buffer supplied by the manufacturer. The Arctic Phosphatase was inactivated by incubation for 5 minutes at 65°C. The dephosphorylated miRNAs were assembled into labeling reactions by the addition of RNA ligase buffer (Roche, supplied by manufacturer) and DMSO (Sigma) to a final concentration of 1x, and 25%, respectively. The miRNAs were denatured at 95°C for 3 minutes, followed by immediate incubation in an ice-water bath for 3 minutes. pCp-Cy3 (for common reference) or pCp-Cy5 (for six size-fractionated samples derived from the infection timecourse) were added to the corresponding denatured miRNA samples on ice to a final concentration of 50µm, followed by the addition of 20 units of T4 RNA ligase (Roche). The ligation reaction was allowed to proceed for 16 hours at 16°C in the dark. 80µl Tris-EDTA was added to the labeled miRNA followed by centrifugation through a microspin-6 column (Bio-Rad) to remove unincorporated label. The labeled RNA in the column flow-through was ethanol-precipitated in the presence of 10mg Glycogen (Roche). The supernatant was aspirated, and miRNA pellets were resuspended in 10µl water. The resuspended Cy-3 and Cy-5-labeled miRNAs were mixed, yielding 20µl of mixed sample. 10µl of 37°C 3x Hybridization mix (Ambion) was added, and the complete hybridization mixture was heated at 95°C for 3 minutes, added to microarrays, and coverslips were carefully dropped onto the arrays. The arrays were hybridized overnight at 37°C in the dark, and washed according to the instructions for the Ambion mirVana™ miRNA Probe Set version 2, except the arrays were dried in an ozone-free environment (see http://cmgm.stanford.edu/pbrown/protocols/Ozone_Prevention.pdf for details). The arrays were scanned with a GenePix scanner and software (Axon 4000a scanner and Genepix analysis software, Molecular Devices) and loaded into the Stanford Microarray Database (http://smd.stanford.edu/) for normalization and data-filtering using the default settings. The heat-map was generated using MEviewer 4.2 freeware (TIGR). In Figure 1C, miRNA families are: ‘miR-17 family’ = hsa-miR-17, hsa-miR-106a, hsa-miR-106b, hsa-miR-20a, hsa-miR-20b, hsa-miR-93, mmu-miR-20b, mmu-miR-106a, mmu-miR-93; ‘miR-18 family’ = hsa-miR-18a, hsa-miR-18b; ‘miR-19 family’ = hsa-miR-19a, hsa-miR-19b; ‘miR-25 family’ = hsa-miR-25, hsa-miR-92.

*High-resolution northern blot* – RNA samples derived from uninfected HFFs, and from HFFs infected with *Toxoplasma* for 24h were resolved through a 10% acrylamide/8M urea/1x Tris/borate/EDTA (TBE) [1x TBE is 89 mM tris base, 89 mM boric acid and 2 mM EDTA] gel, electrotransferred (Hoefer TE77, Amersham) to nylon membrane (Hybond N+, Amersham), and UV-crosslinked (Stratalinker, Stratagene). 500 nmol of each oligonucleotide probe (see Figure S3 for probe sequences) was 5’-end labeled with γ-[^32^P]-ATP (6000 ci/mMol, MP biomedicals) and T4 polynucleotide kinase (New England Biolabs) according to the manufacturer’s instructions; unincorporated γ-[^32^P]-ATP was removed by passage of the reaction mixture through a Micro-Spin 6 column (Bio-Rad). 10ng of 10bp DNA ladder (Invitrogen) was 5’-end [^32^P]-labeled under exchange-reaction conditions for 10 minutes at 37°C [50 mM imidazole (pH 6.4), 12 mM MgCl2, 1mM 2-mercaptoethanol, 50 µM ADP, 10µCi γ-[^32^P]-ATP, 10 units T4 polynucleotide kinase]; unincorporated γ-[^32^P]-ATP was removed by passage of the reaction mixture through a Micro-Spin 6 column. The northern blot was hybridized with [^32^P]-labeled miR-21, miR-17 and U6 oligonucleotide probes at 37°C overnight in oligo hybridization buffer [3x SSC, 0.5% SDS, 1x Denhardt's Solution, 10µg/mL yeast tRNA] washed 3 times for 15 minutes in 2x SSC/0.1% SDS at 42°C, and exposed to a phosphorimager screen (Amersham). U6 snRNA was used as a loading control. Exposed phosphorimager screens were scanned with a Phosphorimager Storm and bands were quantified using phosphorimager analysis software (Molecular Devices).

*Primer-extension analysis –* Total RNA from three biological replicate timecourse experiments were extracted with TRIzol according to the manufacturer’s instructions. 10µg replicate aliquots of each RNA sample were lyophilized in a Speedvac in siliconized 1.7ml microfuge tubes. Lyophilized pellets were stored at -20°C until use. For primer-extension analysis of the *Toxoplasma-*infection time-course (Figure 2A) and *Neospora*-infection time-course (Figure 3A), primer-extensions were performed on three biological replicate total RNA samples extracted from mock- or parasite-infected HFFs at each timepoint (6-hours, 12-hours and 24-hours). Oligonucleotide probes (Figure S3) were 5’-end labeled as described above. 50nM of [^32^P]-labeled oligonucleotide probe (except 5S, see below) was added to each 10µg RNA pellet in 1x PE reaction buffer [50mM Tris-HCL pH 8.3; 75mM KCL, 3mM MgCl_2_], followed by the addition of a 200µM mixture of all four dNTPs (Invitrogen). In the case of 5S primer-extensions, 50nM of [^32^P]-labeled oligonucleotide plus 2µM of cold 5S probe was added to each 10µg RNA pellet in 1x PE reaction buffer, followed by the addition of a 200µM mixture of all four dNTPs. To identify any non-templated artifacts of primer-extension, one primer-extension reaction was performed in parallel for each probe in the absence of template RNA. The primer-extension reaction mixture was heated to 95°C for 5 minutes, slow-cooled in a heat block to 60°C, spun quickly to remove condensation, and added to a second heat block pre-equilibrated to 50°C (Figures 2 and 3), where the samples were allowed to equilibrate to the block temperature for one minute. 20 units of Moloney Murine Leukemia Virus reverse transcriptase (Invitrogen) was added to each mixture and the reactions were allowed to progress for 5 minutes at Tm=50°C. The 6µl reactions were terminated by addition of 4µl of stop mix [45% formamide, 20mM EDTA, 0.05% bromophenol blue, 0.05% xylene cyanol]. After primer-extension, samples were heated to 95°C for 3 minutes, snap-cooled in ice-water, spun down in a microcentrifuge, and resolved by electrophoresis through a 12% acrylamide (Bio-Rad) + 8M urea (Invitrogen) + 1x TBE [89mM Tris-borate pH 8.0, 2mM EDTA] sequencing gel capped at 50 watts for 1 hour. The gel was disassembled, cut, dried, and exposed to a phosphorimager screen for 18-24 hours and scanned. Band intensities were quantified with phosphorimager analysis software.

*pri-miRNA northern blot -* total RNA was isolated from uninfected passage 10 HFFs (‘HFF’ lane), or HFFs infected for 24 hours with *Neospora* (‘HFF + Neo’ lane) or *Toxoplasma* (‘HFF + Toxo’ lane). Extractions were performed in biological duplicate. Poly-A+ RNA was enriched from total RNA using oligo-dT-conjugated beads (Oligotex, Qiagen) according to the manufacturer’s instructions. The bead eluate for each RNA sample was supplemented to a final concentration of 0.3M sodium acetate and was concentrated by ethanol precipitation with 2µl of Pellet Paint (Pierce). The RNA pellet was resuspended in gel loading buffer [11µl water, 8µl 37% formaldehyde, 3µl 100% formamide, 2.5µl 10x MESA (Sigma), 0.5µl 0.125% bromophenol blue] and entire fractions were loaded into individual wells of a 1% agarose/formaldehyde/1x MESA gel, transferred to a nylon membrane (Zetaprobe, Bio-Rad), and crosslinked to the membrane (Stratalinker, Stratagene). Probe fragments were generated by PCR with specific primers using HFF genomic DNA as a template (see Figure S3 for probe sequences). Probe fragments were cloned into pCR2.1 (Invitrogen) and sequenced. EcoRI-digested fragments were gel purified (Qiagen Gel Extraction kit) to serve as templates for probe labeling. Probes were [^32^P]-labeled with the RadPrime kit (Invitrogen); unincorporated label was removed by passage through a Micro-Spin 6 column (Bio-Rad). Membrane bound pri-miRNAs and mRNAs were subject to repeated hybridization with probes specific for *pri-miR-17~92*, *pri-* *pri-miR-106b~25* and *RPS29* (Figure 4). Hybridizations with probes specific for *miR-106a~363*, *MCM7* and *AldoA* were also performed (data not shown). Hybridizations (42°C) and washes (55°C) were performed with Ultrahyb (Ambion) according to the manufacturer’s instructions. To strip the probe from the membrane, 400ml of boiling 0.625% sodium dodecyl sulfate (SDS, Sigma) was removed from heat and immediately added to the membrane. The membrane was immediately placed in this >95°C 0.625% SDS. The membrane in stripping buffer was allowed to cool to room temperature for 30 minutes with gentle agitation. This stripping procedure was repeated sequentially 3 times, and the membrane was exposed to phosphorimager screen overnight to check for efficient removal of the stripped probe prior to subsequent hybridization with a different probe.

*Toxoplasma growth kinetics in miRNA knockdown- or knockout cell lines* – These data were not shown but are referenced in the Discussion section. Individual antisense locked nucleic acid (LNA) oligonucleotides that hybridize to miR-122 (as an unaffected miRNA knockdown control), or the miR-17, miR-18, miR-19 and miR-25 families, were synthesized (Integrated DNA Technologies). Subconfluent NIH 3T3 MEFs or HFFs plated on glass coverslips and/or in tissue culture plates were mock-transfected, or transfected with the individual LNAs listed above, or transfected with mixtures containing all LNAs that hybridized to *miR-17~92*-encoded miRNAs over a concentration range of 1nM to 100nM using Lipofectamine LTX (Invitrogen) according to the manufacturer’s instructions. Transfected NIH 3T3s and HFFs rounded up and detached from the cover slips and flasks at LNA concentrations above 50nM (for all LNAs tried), and therefore 25-35nM LNA was selected as the LNA concentration range for subsequent assays. These transfected cells were infected with *Toxoplasma* and fixed at 12h, 24h, 36h, 48h or 72h post-infection in 4% formaldehyde/1xPBS. A similar series of experiments were performed where primary MEFs harboring *loxP*-flanked *miR-17~92* loci were infected with either wildtype *Toxoplasma* or an engineered *Toxoplasma* strain that facilitates the rapid excision of *loxP*-flanked loci shortly after parasite invasion (A. Koshy, A. Fouts and J. Boothroyd, in preparation); these samples were fixed at the same time points as indicated above. Parasites/vacuole were quantified by fluorescence microscopy. At least 3 independent biological replicates were performed for each condition and each timepoint. No reproducible effects upon parasite invasion, growth, or egress were observed under any LNA transfection condition at any time point, or in experiments with MEFs harboring a conditional knockout of *miR-17~92*.

**References**

1. TODARO GJ, GREEN H (1963) Quantitative studies of the growth of mouse embryo cells in culture and their development into established lines. J Cell Biol 17: 299-313.

2. PULVERTAFT JV (1964) CYTOLOGY OF BURKITT'S TUMOUR (AFRICAN LYMPHOMA). Lancet 1: 238-240.

3. Ventura A, Young AG, Winslow MM, Lintault L, Meissner A et al. (2008) Targeted deletion reveals essential and overlapping functions of the miR-17 through 92 family of miRNA clusters. Cell 132: 875-886.

***Supplemental*** ***Figures***

Figure S1. *miRNA microarray profiling data of Toxoplasma-infected primary human foreskin fibroblasts*. Columns are individual microarrays hybridized with labeled, size-fractionated RNA derived from mock infected HFFs or *Toxoplasma*-infected HFFs at the indicated time-points. Heatmap scale is from -3 to +3, and these values reflect the log_2_-transformed ratios of the hybridization intensities of Cy5-labeled sample/Cy3-labeled common reference for each array. Common reference RNA was a pooled mixture of RNAs from all samples. Data was filtered in SMD according to the default settings, and only spots that were called ‘present’ on 80% of the arrays were included in the heatmap. Log_2_-transformed ratios of the hybridization intensities of sample/common reference for each spot on each array were hierarchically clustered (Euclidean) in MEviewer (MeV4.2; TIGR) by arrays and to genes. Red boxes are miR-17 family members; blue boxes are miR-18 family members. hsa = human, mmu = mouse, rno = rat, ambi = proprietary Ambion probe sequences.

Figure S2. *Northern blot analysis demonstrates that Toxoplasma infection results in increased levels of mature miR-17 family members*. RNA samples derived from uninfected HFFs (- lanes), and from HFFs infected with *Toxoplasma* for 24h (+ lanes) were resolved through a 10% acrylamide/8M urea gel, transferred to nylon membrane, and hybridized to miR-21, miR-17 and U6 oligonucleotide probes (corresponding probes are indicated underneath autoradiographs). The U6 snRNA hybridization is shown as a loading control. Putative pre-miRNA bands and mature miRNA bands are indicated with arrows. A co-electrophoresed 10nt ladder is shown for nucleotide size comparison.

Figure S3. *Probe sequences used in this study*. All oligonucleotides are listed from 5’ to 3’. In ‘notes’ column, target nucleotide positions are provided for all primer-extension probes. The forward and reverse primers used in PCR amplification of the *pri-miR-17~92, pri-miR-106a~363, pri-miR-106b~25, AldoA, MCM7* and *RPS29* probes are shown.
