# Supplementary material for: Toxoplasma gondii Infection Specifically Increases the Levels of Key Host MicroRNAs
Source: PLoS One. 2010 Jan 15;5(1):e8742. doi: 10.1371/journal.pone.0008742 (PMC2806928; doi:10.1371/journal.pone.0008742)
Supplement: Supplementary file 3 [file pone.0008742.s003.doc]

**probe name**	**sequence** (5' to 3')	**Figure**	**Notes**
Hsa U6 snRNA	CACGAATTTGCGTGTCATCCTTGC	S2	nt 90 to 67 of U6 snRNA
miR-21 northern	TCAACATCAGTCTGATAAGCTA	S2	nt 22 to 1 of miR-21
miR-17 northern	CTACCTGCACTGTAAGCACTTTG	S2	nt 23 to 1 of miR-17
5S PE	AGATCGGGCGCGTTCAG	2,3	nt 35 to 19 of 5S rRNA
miR-21 PE	TCAACATCAGTCTGAT	2	nt 22 to 7 of miR-21
miR-17 PE	CTACCTGCACTGTAA	2,3	nt 23 to 9 of miR-17
miR-18 PE	CTATCTGCACTAGAT	2	nt 23 to 9 of miR-18a
miR-19 PE	TCAGTTTTGCATAGA	2	nt 23 to 9 of miR-19a
miR-20 PE	CTACCTGCACTATAA	2	nt 23 to 9 of miR-20a
miR-92 PE	ACAGGCCGGGACAAG	2	nt 8 to 22 of miR-92
pri-miR-17~92 FWD	CAAATATAGATTTGGACG	4	210nt upstream of miR-17
pri-miR-17~92 REV	GAATTCTTTACAGAAGGC	4	210nt upstream of miR-17
pri-miR-106a~363 FWD	GCATAAGATATCGTGACG	4	data not shown
pri-miR-106a~363 REV	CGTCCTCACAGATCCATG	4	data not shown
pri-miR-106b~25 FWD	GTGTGATTACCCAACCTA	4	between miR-93 and miR-25
pri-miR-106b~25 REV	GCCCAGGGCAGCGTCCAG	4	between miR-93 and miR-25
AldoA FWD	TGTGGGCATCAAGGTAGACA	n/a	data not shown
AldoA REV	AAGGTGATCCCAGTGACAGC	n/a	data not shown
MCM7 FWD	GCGTTACATAGCCATGTG	n/a	data not shown
MCM7 REV	CAGAGCAGTGGAAAGGCG	n/a	data not shown
RPS29 FWD	CGTGTCTGTTCAAACCGG	4	
RPS29 REV	TTAGTCCAACTTAATGAA	4	
